# Editorial: Thymic function at single cell resolution

**DOI:** 10.3389/fimmu.2023.1358957

**Published:** 2024-01-08

**Authors:** Martti Laan, Matthieu Giraud, Magali Irla

**Affiliations:** ^1^ Molecular Pathology, Department of Biomedicine, Institute of Biomedicine and Translational Medicine, Faculty of Medicine, University of Tartu, Tartu, Estonia; ^2^ INSERM, Nantes Université, Center for Research in Transplantation and Translational Immunology, UMR 1064, Nantes, France; ^3^ Centre d’Immunologie de Marseille-Luminy (CIML), CNRS, INSERM, Aix-Marseille Université, Marseille, France

**Keywords:** thymus, thymic epithelial cells (TEC), T cells, single cell RNA-seq (scRNA-seq), thymocyte

The thymus is responsible for the generation of a self-tolerant T-cell repertoire capable of recognizing pathogens or cancer cells. The different steps of T-cell development and selection from early T-cell progenitors are controlled by instructive cues provided by the complex network of thymic epithelial cells (TECs) and dendritic cells (1). Recent advances in molecular biology based on single-cell RNA-seq (scRNA-seq) and ATAC-seq have recently revealed a previously unsuspected high degree of heterogeneity in medullary TECs (mTECs) (2). However, much remains to be elucidated to improve our understanding of thymic function. Using transcriptomic approaches, this Research Topic contributes to unraveling several important aspects such as the development of TECs in the embryo or the effects of irradiation-induced stress on TECs. It also provides new findings on the identification of TEC populations and the metabolism of developing T cells in the human thymus.

The review of Matsumoto et al. discusses how single-cell transcriptional profiling of TECs have revealed an unanticipated level of heterogeneity with the discovery of “mimetic” mTEC populations (2). They correspond to later stages of mTEC differentiation and mirror extrathymic cell types. It is becoming clear that the full load of tissue-restricted self-antigens (TRAs) expressed and presented by mTECs to developing thymocytes comprise Aire-induced TRAs and the high diversity of “mimetic” TRAs. In addition to its direct role on TRA expression, Aire has been shown to drive the maturation of some “mimetic” mTEC populations, highlighting an additional indirect effect of Aire on TRA expression. Thus, scRNA-seq approaches have greatly overhauled our view of mTEC heterogeneity and TRA expression. Importantly, combination of deeper scRNA-seq strategies with scATAC-seq in human and mouse models will undoubtedly extend our understanding of mechanisms underlying the development of mimetics mTECs and thus TRA expression in the thymic medulla.

How TECs develop and differentiate into mature mTECs and cortical TECs (cTECs) is a central question that remains largely unresolved. Identification of TEC progenitors and molecular pathways that modulate their differentiation into mTECs and/or cTECs would allow control of m/cTEC repopulation *in vivo*. It will also enable tighter control of direct differentiation of patient-derived induced pluripotent stem cells into mature TECs and thymic organoids. These organoids with fine-tuned mTEC and cTEC populations would support thymocyte development and the generation of functional regulatory T cells. Whereas the classical model of TEC differentiation relies on the existence of a bi-potent progenitor that gives rise to cTECs and mTECs (3), it has been refined by the work of Farley et al. The authors revealed the existence of mTEC and cTEC-fated sublineages in the embryonic thymus. The corresponding progenitors are already present at the earliest stages of thymus organogenesis and show mTEC- vs cTEC-fate plasticity.

Horie et al. analyzed the impact of acute irradiation on mTEC heterogeneity and TRA expression. Irradiation, used to treat cancer, causes thymic involution, characterized by a severe reduction of thymocytes and TECs (4, 5). The authors show that after total body irradiation, thymocytes decrease until day 4, and are restored on day 10 whereas TECs decrease until day 6 with a maximum recovery on day 15. They therefore assessed TEC recovery 15 days after irradiation by scRNA-seq and found that Aire^+^ mTEC, and their progeny, i.e. late-Aire, post-Aire, and tuft-like mTEC, were more affected than transit amplifying-TEC progenitors, CCL21^+^ mTECs and cTECs. They propose that this reduction in Aire^+^ mTEC, and their progeny is likely due to cell death of these populations and/or a repression of mTEC differentiation. Moreover, TRA expression was slightly reduced in Aire^+^ and late-Aire mTECs and intriguingly a subcluster of Aire^+^ mTECs expressing CCL25 and TRA was severely decreased. Although longer-term effects on TECs remain to be investigated, this study suggests that irradiation may disrupt central tolerance induction.

Although recent advances in scRNA-seq methodology have broadened our understanding in gene expression patterns and helped to discover new mTEC populations (6), there is still a clear need for better cellular surface markers for flow cytometry (FC)-based TEC quantification, sorting, and *ex vivo* experimental studies. This is particularly true in humans, where defining TEC populations with markers derived from mice were not optimal (7). In line with this, by using FC-based based surface marker screening, Haunerdinger et al. propose a novel combination of surface markers for human TECs (Pdpn^high/int^EpCAM^high/int/low^) with further subdivision into mTECs (CD49f^int/low^CD200^+^) and cTECs (CD49f^+^CD200^-^). In addition, the authors validated the proposed markers by RNA-seq of the accordingly FC-sorted cells as well as by immunostaining, providing a novel identification strategy for human TEC populations.

During their coordinated migration through different thymic compartments, developing thymocytes go through well-characterized developmental stages from double-negative (DN) to immature single positive (iSP) to double positive (DP) and finally to single positive (SP) CD4^+^ or CD8^+^ cells. Although the accompanying changes in gene expression have been characterized in detail (8, 9), the metabolic status of thymocytes during their maturation is largely unknown. Accordingly, the study by Sun et al. uses extracellular flux analysis together with transcriptomic profiling and shows that the indicators of glycolysis and oxidative phosphorylation as well as related transcriptional pathways peak at the DN and iSP stages followed by a massive drop at the DP stage and a partial recovery at the SP stages. The authors also show that these metabolic changes are conserved between mice and humans, do not require support from complex multicellular stroma and are largely independent of TCR rearrangement. The critical factors triggering these changes, however, remain to be determined in future studies.

Research gathered in this topic brings great progress in the study and understanding of thymic cell heterogeneity and function on T cell development. It also clearly highlights the unprecedented power of the novel molecular biology techniques to uncover thymic mysteries, opening up fine prospects for future thymus research.

## Author contributions

ML: Writing – original draft, Writing – review & editing. MG: Writing – original draft, Writing – review & editing. MI: Writing – original draft, Writing – review & editing.

## References

[1] Irla M. Instructive cues of thymic T cell selection. Annu Rev Immunol (2022) 40:95–119. doi: 10.1146/annurev-immunol-101320-022432 35471838

[2] Michelson DA Hase K Kaisho T Benoist C Mathis D. Thymic epithelial cells co-opt lineage-defining transcription factors to eliminate autoreactive T cells. Cell (2022) 185:2542–58.e18. doi: 10.1016/j.cell.2022.05.018 35714609 PMC9469465

[3] Rossi SW Jenkinson WE Anderson G Jenkinson EJ. Clonal analysis reveals a common progenitor for thymic cortical and medullary epithelium. Nature (2006) 441:988–91. doi: 10.1038/nature04813 16791197

[4] Lopes N Vachon H Marie J Irla M. Administration of RANKL boosts thymic regeneration upon bone marrow transplantation. EMBO Mol Med (2017) 9:835–51. doi: 10.15252/emmm.201607176 PMC5452038 28455312

[5] Kinsella S Dudakov JA. When the damage is done: injury and repair in thymus function. Front Immunol (2020) 11:1745. doi: 10.3389/fimmu.2020.01745 32903477 PMC7435010

[6] Kadouri N Nevo S Goldfarb Y Abramson J. Thymic epithelial cell heterogeneity: TEC by TEC. Nat Rev Immunol (2019) 20:239–53. doi: 10.1038/s41577-019-0238-0 31804611

[7] Gotter J Brors B Hergenhahn M Kyewski B. Medullary epithelial cells of the human thymus express a highly diverse selection of tissue-specific genes colocalized in chromosomal clusters. J Exp Med (2004) 199:155–66. doi: 10.1084/jem.20031677 PMC2211762 14734521

[8] Kernfeld EM Genga RMJ Neherin K Magaletta ME Xu P Maehr R. A single-cell transcriptomic atlas of thymus organogenesis resolves cell types and developmental maturation. Immunity (2018) 48:1258–70.e6. doi: 10.1016/j.immuni.2018.04.015 29884461 PMC6013397

[9] Park J-E Botting RA Conde CD Popescu D-M Lavaert M Kunz DJ. A cell atlas of human thymic development defines T cell repertoire formation. Science (2020) 367(6480):eaay3224. doi: 10.1126/science.aay3224 32079746 PMC7611066

